# Pattern of induced estrus and conception rate following Ovsynch and Ovsynch based gonadotropin-releasing hormone treatments initiated on day 6 of estrous cycle in repeat breeding crossbred cows

**DOI:** 10.14202/vetworld.2016.342-345

**Published:** 2016-04-02

**Authors:** N. Ahmed, D. Kathiresan, F. A. Ahmed, K. Lalrintluanga, P. Mayengbam, J. M. Gali

**Affiliations:** 1 Department of Animal Reproduction, Gynaecology and Obstetrics, College of Veterinary Sciences and Animal Husbandry, Central Agricultural University, Aizawl, Mizoram, India; 2 Department of Veterinary Physiology and Biochemistry, College of Veterinary Sciences and Animal Husbandry, Central Agricultural University, Aizawl, Mizoram, India

**Keywords:** accessory corpus luteum, conception rate, estrus response, Ovsynch, repeat breeding

## Abstract

**Aim:**

The aim was to evaluate the estrus response, incidence of accessory corpus luteum formation and fertility following different hormonal protocols in repeat breeding crossbred cows.

**Materials and Methods:**

This study was carried out on 24 repeat breeding crossbred cows allotted into four groups. Cows of Group I was not given any treatment, Group II was treated with gonadotropin-releasing hormone (GnRH) injection on day 6 post-estrus, Group III was treated with Ovsynch protocol, and Group IV was treated with Ovsynch based GnRH treatment. Estrus responses such as duration, onset, percentage, and intensity of estrus were recorded during the study. The incidence of accessory corpus luteum was recorded per rectally on day 7 after first and additional GnRH of Ovsynch treatment. The conception rate for all groups was calculated by the absence of estrus and on day 45 after artificial insemination (AI) per rectum. Serum samples were collected at AI and day 12 post-AI in Group I and II. Serum samples were also collected at GnRH, Prostaglandin F_2α_ (PGF_2α_), timed AI (TAI) and day 12 post-TAI in Group III and IV.

**Results:**

Ovsynch and Ovsynch based GnRH treatments are resulted in 100.00% induction of estrus after the PGF_2α_ injection. Onset of induced estrus after the PGF_2α_ injection for Group III and IV was recorded as 48.750±0.713 and 51.472±1.989 h, respectively, and it was not significant. There was no significant difference in duration of estrus among the groups. The incidence of intermediate estrus intensity was found to be highest. All the cows showed the incidence of formation of accessory corpus luteum subsequent to GnRH treatment on day 6 of the estrous cycle in Group II, III, and IV. The conception rate was 0.00%, 16.67%, 50.00%, and 50.00% in Group I, II, III, and IV, respectively.

**Conclusion:**

Ovsynch and Ovsynch based GnRH treatments initiated on day 6 of estrous cycle capable of responding with a higher percentage of ovulation and formation of accessory corpus luteum which helped in higher conception rate over single post-AI GnRH treatment in repeat breeders. These treatments responded with better estrus response but did not significantly improve estrus intensity.

## Introduction

The dairy industry is the mainstay of Indian agricultural growth. Repeat breeding is a major constraint with the incidence ranges from 5.5% to 33.33% [1]. Repeat breeding in dairy cows is associated with estrus detection error, endocrine dysfunctions, ovulation defects, uterine infection, gamete quality, etc., and therefore, poor fertilization rate and/or early embryonic death [2]. Therefore, Ovsynch protocol has been developed which synchronizes estrus and ovulation to overcome these constraints. However, Ovsynch also has some drawbacks such as premature estrus, reduced ovulation to first gonadotropin-releasing hormone (GnRH), and inconsistent conception rate [3]. Studies revealed that the effectiveness of Ovsynch is greatest when treated cows ovulate to the first GnRH. Ultrasonographic studies also opined that synchronization of follicular wave emergence can be successfully achieved by administering GnRH on day 5 or 6 of estrous cycle resulting ovulation of first wave dominant follicle with the formation of accessory corpus luteum in crossbred cows [4,5].

Many methods have been tried to increase conception rate by enhancing endogenous progesterone level as lower than the normal rise and lower total progesterone concentration have been reported in repeat breeder cows. This can be achieved by inducing the formation of accessory corpus luteum, which can be obtained by GnRH treatment on day 4-6 post-artificial insemination (AI) [6,7].

The present study was therefore aimed to evaluate the estrus induction and fertility response by using Ovsynch and Ovsynch based gonadotropin-releasing hormone treatments initiated on day 6 of estrous cycle in repeat breeding crossbred cows.

## Materials and Methods

### Ethical approval

The prior approval from the Institutional Animal Ethics Committee was obtained for use of farmers’ animals in this study.

### Selection of animals

The study was carried out from November 2014 to June 2015. 24 apparently healthy crossbred cows having a history of repeat breeding were included for the study. The cows were selected from four nearby villages of College of Veterinary Sciences and Animal Husbandry, Aizawl, Mizoram. The cows were screened twice at 10 days interval through gyneco-clinical examination. Cows having a corpus luteum on rectal examination and no palpable abnormalities of the genital tract were considered as cyclical animals and subjected to the study. The cows were maintained under standard feeding and good management system according to milk yield.

### Treatment regimens

About 24 repeat breeding crossbred cows were randomly and equally divided into four groups *viz*.

#### Group I (control)

All the cows were observed for the signs of estrus and AI was performed during estrus by AM/PM rule.

#### Group II

Cows were observed for signs of estrus, and AI was done during observed estrus by AM/PM rule. In addition, the cows were injected with a dose of 10 µg of buserelin acetate-GnRH analog (injection gynarich, 2.5 ml, Intas Pharmaceutical) intramuscular (I/M) on day 6 after estrus.

#### Group III

In this protocol, Ovsynch treatment as described by Pursley *et al*. [8] was initiated on day 6 of estrous cycle (day 0) after detection of estrus which consisted of I/M injection of 10 µg of GnRH analog on day 0, 500 µg of Cloprostenol-prostaglandin F2α (PGF_2α_) analog (injection pragma, 2 ml, Intas Pharmaceutical) 7 days later (day 7), another 10 µg of GnRH analog 48 h after PGF_2α_ injection (day 9), and timed AI (TAI) at 16-18 h after second GnRH injection (day 10).

#### Group IV

In this group, all the experimental animals were treated alike Group III. In addition, they were injected with a dose of 10 µg of GnRH analog I/M on day 6 after the second GnRH injection of Ovsynch treatment.

### Estrus response

Estrus was detected on the basis of behavioral, physical, and gynecological examination. The gynecological examination was carried out 24 h after the PGF_2α_ injection and then at every 12 h to confirm estrus in Group III and IV crossbred cows. Estrus response in percentage was calculated as a number of cows expressed estrus. The onset of estrus was calculated in hours (h) from the time of PGF_2α_ administration to the time of the first appearance of estrus signs in Group III and IV. Duration of estrus was calculated from the time of the first appearance of estrus signs to the time of disappearance of estrus signs (h). The intensity of estrus was assessed based on the scorecard described by Rao and Rao [9] in crossbred heifers with slight modifications.

### Conception rate

The conception rate was calculated for each group by the following equation:


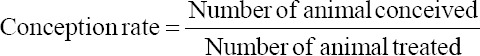


### Serum progesterone analysis

Serum samples were collected at AI and day 12 post-AI in Group I and II. Similarly, Serum samples were collected at the time of GnRH injection (day 0), PGF_2α_ injection (day 7), TAI (day 10), and on day 12 post-TAI in the case of Group III and IV. Serum progesterone concentration was analyzed by progesterone ELISA kit (Enzo Life Science^®^). The sensitivity of the assay was 8.57 pg/ml.

### Statistical analysis

The data on estrus response (t-test) and conception rate (Chi-square test) were analyzed statistically using SPSS software.

## Results and Discussion

### Estrus response

In this study, Ovsynch treatment was initiated on day 6 of estrous cycle and all the cows (100%) responded to Ovsynch and Ovsynch based GnRH treatments with expression of behavioral and physical signs of estrus within 48.750±0.713 and 51.472±1.989 h, respectively, from the time of PGF_2α_, which was in accordance with the earlier observations in repeat breeder cows and buffaloes [10,11]. Whereas, Kasimanickam and Carabă and Velicevici [12,13] recorded much lower estrus induction response of 17.70 and 63%, respectively. In this study, the Ovsynch treatment was initiated on known day, i.e., the 6^th^ day of estrous cycle and the higher percentage of estrus response probably due to the presence of a functional corpus luteum of 12-13 days and an accessory corpus luteum of 7 days age at the time of PGF_2α_ injection secreting higher endogenous progesterone which was associated with greater probability of luteolysis and progesterone level dropped down to sub-optimal level (Table-1) which helped in onset of behavioral and physical signs of estrus. The time taken for onset of estrus in Ovsynch and Ovsynch based GnRH treatments was in line with the intervals reported earlier [10,14]. However, Chaudhary *et al*. [15] reported a higher interval to onset of estrus in Ovsynch treated crossbred cows. There was no significant difference regarding duration of estrus among the different groups, which concurred with the findings of Bhat and Bhattacharyya [16]. The incidence of moderate estrus was significantly higher, and the intense and weak estrus was significantly lower in all the groups irrespective of different regimes (Table-2), which was an agreement to that of Velladurai *et al*. [14] and Alyas *et al*. [17].

**Table-1:** Serum progesterone concentration at different days of treatment/AI in repeat breeding crossbred cows.

Treatment protocol	Progesterone concentration (ng/ml) at				F value

	GnRH	PGF_2α_	AI/TAI	Day 12 post-AI	
Control	-	-	0.252^bC^±0.017	3.171^aC^±0.295	97.587**
GnRH treatment on day 6 post-estrus	-	-	0.350^bB^±0.025	4.690^aAB^±0.533	66.126**
Ovsynch	1.421^Bc^±0.114	3.647^b^±0.102	0.464^dA^±0.026	4.132^aBC^±0.087	387.886**
Ovsynch based GnRH treatment	1.878^Ac^±0.315	3.732^b^±0.077	0.358^dB^±0.011	5.482^aA^±0.403	116.657**
F value	14.823**	0.444^NS^	17.528**	6.975**	

** Significant at p≤0.01, Means bearing different superscripts in the capital and small letter differ significantly between columns and rows respectively. GnRH=Gonadotropinreleasing hormone, AI=Artificial insemination

**Table-2:** Natural and induced estrus response in repeat breeding crossbred cows of treatments and control group.

Treatment protocol	Onset of estrus (h)	Duration of estrus (h)	Intensity of estrus (%)			Conception rate (%)

			Intense	Intermediate	Weak	
Control	-	22.042±0.949	16.67	50.00	33.33	0.00
GnRH treatment on day 6 postestrus	-	21.458±1.100	33.33	33.33	33.33	16.67^b^
Ovsynch	48.750±0.713	21.083±0.787	16.67	66.67	16.67	50.00^a^
Ovsynch based GnRH treatment	51.472±1.989	20.070±0.863	33.33	50.00	16.67	50.00^a^

Significant at p≤0.01, values bearing different superscripts differ significantly. GnRH=Gonadotropin-releasing hormone

### Incidence of accessory corpus luteum

In the present study, 100% incidence of formation of accessory corpus luteum was recorded to the GnRH injections given on day 6 of the estrous cycle in all the treatment groups. The findings were in agreement with that recorded by early researchers in lactating dairy cows [4,18]. Foster *et al*. [19] also opined that the administration of GnRH on day 5 or 6 of estrous cycle overrides the negative feedback of progesterone on the anterior pituitary, thereby allowing the secretion of both luteinizing hormone and follicle-stimulating hormone, resulting in ovulation of the dominant follicle, and subsequent formation of a corpus luteum. Satheshkumar *et al*. [5] documented the follicular development pattern through ultrasonographic studies in Jersey crossbred cattle and opined that the properties of first wave dominant follicle was consistent and highly predictable than the dominant follicle of subsequent waves and also confirmed that the dominant follicle of the first wave was in growing phase with >9 mm in diameter by day 6 of estrous cycle and the 100% ovulation to GnRH. The presence of accessory corpus luteum resulted in higher endogenous progesterone secretion which ultimately prevented the premature estrus induction during Ovsynch protocol as well as helped in greater effectiveness of Ovsynch treatment.

### Conception rate

The conception rate was significantly higher in groups treated with Ovsynch and Ovsynch based GnRH treatments than the other groups (Table-2). These results were in agreement with the previous reported in repeat breeder cows [10,20]. Present findings were lower than those recorded by others [21,22]. Studies revealed that the initiation of Ovsynch treatment on different days of estrous cycle influenced its effectiveness [23]. Ultrasonographic studies opined that initiation of Ovsynch on day 6 of estrous cycle induced ovulation of dominant follicle of growth phase and formed an accessory corpus luteum, resulted in higher progesterone concentration at PGF_2α_ injection (Table-1) of Ovsynch treatment which was associated with greater probability of luteolysis and conception rate [24]. Moreover, Ovsynch initiated on day 6 of estrous cycle could emergence a new follicular wave in crossbred cows and presence of a dominant follicle at the time of second GnRH (day 9) of Ovsynch treatment resulted in cent per cent ovulation of the present follicle [25,26] and formation of corpus luteum which secreted and maintained higher progesterone during critical stage of embryonic development. Additional GnRH in Ovsynch based GnRH treated group helped in better conception rate by additional progesterone secretion through accessory corpus luteum (Table-1).

## Conclusion

Ovsynch and Ovsynch based GnRH treatments initiated on day 6 of estrous cycle capable of responding with a higher percentage of ovulation and formation of accessory corpus luteum which helped in higher conception rate over single post-AI GnRH treatment. These treatments responded with better estrus response. However, these changes did not significantly improve the estrus intensity in repeat breeding crossbred cows.

## Authors’ Contributions

NA, DK, FAA, KL, and PM designed the study. The experiment was done by NA, DK, FAA, KL, and PM, while laboratory work was done by NA and JMG. All the authors participated in data analysis, draft, and revision of the manuscript. All authors read and approved the final manuscript.
